# Understanding Technology-Induced Value Change: a Pragmatist Proposal

**DOI:** 10.1007/s13347-022-00520-8

**Published:** 2022-04-15

**Authors:** Ibo van de Poel, Olya Kudina

**Affiliations:** Section Ethics and Philosophy, Department of Values, Technology & innovation, School of Technology, Policy & Management, TU Delft, Jaffalaan 5, 2628 BX Delft, The Netherlands

**Keywords:** Technology, Value, Value change, Pragmatism, Dewey, Technomoral change

## Abstract

We propose a pragmatist account of value change that helps to understand how and why values sometimes change due to technological developments. Inspired by John Dewey’s writings on value, we propose to understand values as evaluative devices that carry over from earlier experiences and that are to some extent shared in society. We discuss the various functions that values fulfil in moral inquiry and propose a conceptual framework that helps to understand value change as the interaction between three manifestations of value distinguished by Dewey, i.e., “immediate value,” “values as the result of inquiry” and “generalized values.” We show how this framework helps to distinguish three types of value change: value dynamism, value adaptation, and value emergence, and we illustrate these with examples from the domain of technology. We argue that our account helps to better understand how technology may induce value change, namely through the creation of what Dewey calls indeterminate situations, and we show how our account can integrate several insights on (techno)moral change offered by other authors.

## Introduction

Philosophers have often understood morality as eternal and unchangeable, but recently, there has been a growing attention for moral change (e.g., Appiah, 2010; Baker, 2019; Calman, 2004; Eriksen, 2020; van der Burg, 2003). Here, we are interested how morality, and more specifically values, might change due to technological developments. Recently, several authors have suggested that morality is at least in part shaped by technology (Swierstra & Rip, 2007; Verbeek, 2011). Technology has, for example, created new moral problems: the proliferation of genetically modified food has raised concerns about global justice and responsibility, while the norms of food safety and equitable access to this technology have subsequently undergone review. Assisted reproduction technologies have defied previously existing biological horizons, enabling aged, infertile, or same-sex couples to have children. This has not only redefined what it means to be human but also fostered novel normative expectations regarding procreative rights and liberties. Or to give a more mundane example, consider how glass office doors enable the expectation of transparency in the professional setting, while simultaneously reducing the value of privacy and internalizing a surveillance gaze within employees. Technologies, thus, while being the fruits of human creativity, manifest not merely as neutral tools but also as productive elements in co-shaping how people perceive the world, each other, and themselves. Moreover, this “co-evolution of technology and society […] does not halt at the door of morality” (Swierstra et al., 2009, 120).

Several authors have written on the ethical dimension of technology, considering how technology embeds values, inspiring human actions and understandings (Parens, 2015); how it reduces moral engagement and relations (Turkle, 2007); and how it can provide moral insights for people (Wallach & Allen, 2009). Moreover, technology can aid in changing the values with which we approach and evaluate it, co-shaping our moral perceptions and choices (Verbeek, 2011) and redefining the meaning of our values throughout technological adoption and use (Van de Poel, 2021; Kudina, 2019).

The thinking about the relation between technology and values has in the philosophy of technology evolved from the view, held by philosophers like Ellul (1964) and Heidegger (1962), that Technology, with a big T, fosters values like efficiency and an instrumental view of reality, towards the idea that values can shape the design of specific technologies (Winner 1980)), and more recently, the view that technology and value co-shape each other (Swierstra, 2013; Van de Poel, 2020). Meanwhile, in moral philosophy and applied ethics, there has been an increased recognition that moral values are not static but dynamic and may change over time (Appiah, 2010; Baker, 2019; Calman, 2004; Eriksen, 2020; van der Burg, 2003). Several authors have recently drawn attention to the role of technology in moral change (Swierstra et al., 2009, Swierstra, 2013, Nickel, 2020, Nickel et al., (2022), van de Poel, 2021).

Value change is an important phenomenon for a number of reasons. First, it helps us to better philosophically understand the relation between technology and morality. Second, value change should be accounted for if we aim at pro-actively integrating values of moral importance in the design of new technologies as advocated in approaches like value-sensitive design (Friedman & Hendry, 2019) or responsible innovation (Owen et al., 2013). Third, value change may sometimes be required to better address new moral problems we are confronted with (as our account will suggest).

The aim of this paper is to systematically develop a pragmatist account of value change and to explore how such an account may help to better understand how technology may trigger value change. We are certainly not the first who use pragmatism to understand how technology may trigger moral change. Particularly, the scholarship on technomoral change has been inspired by a pragmatist view on morality, where morality is conceived as established moral routines, which may be disrupted by new technologies (Keulartz et al., 2002; Swierstra, 2013). In this contribution, our focus is more specifically on values, and we draw in detail on the pragmatist philosopher John Dewey to develop a pragmatist account of values and value change, in order to show how it may be productive for understanding value change in relation to technology. In our understanding, value change is primarily triggered by technology if and when technological developments lead to new indeterminate situations which trigger new inquiries. We try to show in detail how such new inquiries can lead to different types of value change, which we describe respectively as value dynamism, value adaptation, and value emergence.

The paper starts with a brief presentation of John Dewey’s moral philosophy. We will argue that, for Dewey, activities like valuing and evaluation are more central than values, but that a pragmatist account may nevertheless understand values as *evaluative devices that help us to analyze and diagnose morally problematic situations*. We will then introduce a conceptual framework that — following Dewey — distinguishes between three levels at which values play out, i.e., as immediate valuing, in inquiry, and as generalized values. We will use this framework to distinguish different dynamics of value change that we will denote as *value dynamism*, *value adaptation*, *and value emergence*. We illustrate these dynamics with a number of examples from the domain of technology and argue that our account helps to better understand how technology may trigger value change by comparing it with a number of existing accounts from the literature on (techno)moral change.

## Dewey on Values

Dewey was a prominent American thinker at the turn of the twentieth century, commonly referred to as an educator, policy-maker, psychologist, and philosopher. We would like to draw attention to Dewey’s contribution in the field of moral philosophy grounded in his pragmatist practice-oriented thought. In his rich scholarship, Dewey gave much attention to the nature, function, and character of values as both representing the past experiences of people and guiding their future action. Pivotal here is the consideration of the environmental embedding of values in the socio-material practices of people, understood as an infrastructure that both enables values and challenges their meaning. Values thus appear as both relational to the context and giving guidance to future situations.

In Dewey’s pragmatist thought,^1^values do not refer to mere preferences and likings of people; instead, he considers such emotive reactions as the first step in understanding and realizing values. Values, rather, are a reflective product of a judgement of the situation at hand, a working solution that resolves tensions that a confronting situation presents. Because values are weighted and considered, they bare legitimacy over future similar situations, being a shortcut to past deliberations. Such a practical nature of values as orienting judgement devices underpins their simultaneously historical and dynamic, hermeneutic and normative character.

Dewey rejects the idea that values exist in isolation (Dewey, 1939, 55–57). Instead, we should, according to him, always understand values in relation to their conditions (i.e., what brings the value about) and consequences (what is brought about by the value). This relational idea of values explains why Dewey rejects the often-made distinction between instrumental and final (or intrinsic) values. Dewey does not deny that we can analytically make such distinctions, nor that they sometimes have moral significance, but for him, every end is provisional, and, historically as well as causally, a means to a further end (e.g., Dewey, 1939, 26 and 43). He therefore prefers to talk about “ends in view” (Dewey, 1922a, 1939). Moreover, he believes that we cannot establish the desirability of a certain end (in view) without considering the means that are needed to achieve that end (in view) (e.g. Dewey, 1939, 31).

Dewey’s philosophy of value is primarily a philosophy of what he calls valuation. Dewey sees valuation as proceeding in two stages (Dewey, 1922a, 1938, 1939). The first stage is one of immediate, almost instinctive, or motor-sensory, reaction to a situation. He calls this stage valuing or prizing, and he describes it with such terms like “direct,” “impulsive,” “a tendency,” and “behaviouristic.” The second stage he calls evaluation or appraisal. At this stage, rather than directly or immediately valuing something, one asks the question of whether one *ought* to value something. As Dewey says, “The fact that something is desired only raises the question of its desirability; it does not settle it” (Dewey, 1929a, 260). Raising and answering questions about desirability means that one engages in value judgements, and Dewey holds that such value judgements may result in new values (e.g., Dewey, 1918, 1922b). While he does not deny that value judgements may in part be guided by existing values (as we will explain in more detail below), he firmly believes that value judgements may create new values that did not as such exist before. He, for example, writes “… doubtful values exist which are determined to existence through judgment and only through judgment” (Dewey, 1922b, 334–335).

In Dewey’s writing, we can find three distinct, but related notions of value.^2^ The first notion is that of *direct or immediate value*, i.e. value as the result of what he calls valuing or prizing, and which is determined by our immediate reaction to a certain situation. He believes that philosophically, there is little to be said about this first type of value: “Of immediate values as such, values which occur and which are possessed and enjoyed, there is no theory at all; they just occur, are enjoyed, possessed; and that is all” (Dewey, 1929b, 403). In Dewey’s view, value as immediate experience just occurs and is not open to rational discussion or deliberation.

The second notion of value that can be found in Dewey is that of value *as the result of judgement*. Dewey admits that value judgements exist that “merely report, describe, list and classify” what we already know, for example that health is good. He is, however, mainly interested in “another kind of judgement, that here called valuation, which is concerned with estimating values not in existence and with bringing them into existence …” (Dewey, 1922b, 332). It should be noted that value for Dewey here refers not so much to an abstract notion, like health, that we can use to evaluate state-of-affairs but rather to something that is brought about in the world by our actions. Moreover, for him, a value judgement, or a practical judgement as he calls it elsewhere, is itself not only a mere judgement (“we should do x”) but also involves the action (i.e., the doing of x) that is recommended by the judgement as well (see e.g., Dewey, 1915). It is for this reason that he says that the value is brought into existence by the judgement, and that value judgement can bring new values into existence (see also Dewey, 1918).

The third notion of value that we can find in Dewey’s writings is that of value as abstract notion, or moral rule, based on previous judgements and guiding future judgement. As he writes, “Generalized ideas of … values undoubtedly exist. … Similar situations recur; desires and interests are carried over from one situation to another and progressively consolidated. A schedule of general ends results, the involved values being “abstract” in the sense of not being directly connected with any particular existing case … “ (Dewey, 1939, 44). He sees these values as hypotheses that are tested out in new situations: “these general ideas are used as intellectual instruments in judgement of particular cases … tools that direct and facilitate experimentation … while they are also developed and tested by the results of their application in these cases” (Dewey, 1939, 44).

Dewey thus employs three notions of value that may be summarized as (1) value as (immediate) experience, (2) value as a result of judgement (and action), and (3) value as an abstract hypothesis based on earlier experiences. In the next section, where we propose our own pragmatist account of value and value change, we will propose to foreground the third meaning. For now, and for the aim of understanding Dewey’s theory of valuation, it is important that Dewey seems to see these different meanings of value as connected in the sense that they correspond to different phases in the process of inquiry and judgement.

Dewey uses the term “inquiry” to refer to a general process^3^ of investigation and deliberation that can be both more practical as well as more theoretical or scientific. He takes inquiry to have certain general characteristics and a general structure, independent of the specific topic of inquiry. We will highlight the characteristics that are particularly important for understanding how the various understanding of value may surface in inquiry.

For Dewey, inquiry always starts with what he calls an *indeterminate situation*. An indeterminate situation is a situation that is somehow unsettling, incomplete, or felt as unpleasant. Importantly for Dewey, it is not a problematic situation because in a problematic situation, we already know what the problem is. Turning an indeterminate situation into a problematic one is for Dewey the first step of inquiry.

Inquiry, according to Dewey, is aimed at resolving the initial indeterminate situation. This resolution is not just the (intellectual) proposal of a solution but involves the exercising of that solution as well. Inquiry, in other words, is *transformative.* However, this transformation might fail in the sense that it does not successfully transform the initial indeterminate situation into a determinate one. Therefore, inquiry is always *experimental* according to Dewey. It involves trying out certain solutions, and trying again when tried solution fails. This trying out is, however, not just trial-and-error but is guided by *intelligence*. We try out solutions that we have reason to believe to work, for example because they are based on rules that have worked in similar situations before. Such rules for Dewey have the status of hypotheses. Moreover, they are not just applied to a new situation, but rather we reinterpret them in the light of the new situation. In this sense, inquiry has a clearly hermeneutic component in which we interpret new situations in the light of previous experiences and knowledge while at the same time transforming our knowledge and experiences.

With his picture of inquiry in mind, we can see how the three different notions of value in Dewey’s writings connect to different phases of inquiry. Value as immediate experience typically precedes inquiry and due to its direct experiential nature, it is itself not open for further inquiry. Nevertheless, immediate value may involve a sense of unease, something undesirable or unsettling and so set off a process of inquiry. In the process of inquiry, then, values in the third meaning, i.e., as abstract hypotheses based on earlier experiences, do play a role. The result of inquiry is, as we have seen, according to Dewey, a transformation of the real world and this transformation may involve the bringing into existence of a new value, and here, value in the second sense as described above is at play.

## A Pragmatist Account of Values

We will build on Dewey’s account of valuation and values in order to develop our own pragmatist account of value change. In doing so, we do not aim to be consistent with all of Dewey’s ideas. In particular, we propose to disambiguate his notion of value and to focus on one specific meaning of value. We will then describe how this specific notion of value plays a role in evaluation and inquiry, and will develop a notion of value change and distinguish different kinds of value change.

As we have seen, in Dewey’s writings, three notions of value can be found. For the pragmatist account of value change that we develop here, we propose to foreground the third notion that we described above. Henceforth, we will understand values as *evaluative devices that carry over from earlier experiences and are (to some extent) shared in society.*

Let us explain the core elements of this characterization of values.

Values are *evaluative*. In moral philosophy, often a distinction is made between the evaluative and the deontic part of the normative and values are seen as belonging to the evaluative part (e.g., Zimmerman, 2015). They evaluate states-of-affairs (or other things that are being evaluated) in terms of goodness; whereas deontic statements address the rightness of actions. This also means that values are not directly action-guiding, although they may suggest certain actions. Dewey also seems to subscribe to the evaluative-deontic distinction. In “Three Independent Factors in Morals,” he writes “There is an intrinsic difference, in both origin and mode of operation between objects which present themselves as satisfactory to desire and hence good, and objects which come to one as making demands upon his conduct which should be recognized. Neither can be reduced to the other” (Dewey, 1930, 319).

Values are *devices* or tools. This is what makes our account pragmatist. The idea that values are devices or tools (for evaluation) very much fits Dewey’s philosophy and pragmatism more generally. Rather than seeing values as eternal truths, we conceive of them as human constructs or tools that fulfil a number of *functions*, like for example helping to evaluate states-of-affairs or helping to discover what is morally at stake in a certain situation. Below, we will discuss in more detail the various functions that values can fulfil in evaluation and inquiry.

Values *carry over from earlier experiences*. In line with Dewey’s philosophy of valuation, we conceive of values not just as the standards of evaluation, but also as the outcome of evaluation and judgement. In evaluation, we use values that carry over from earlier judgements as hypotheses. These hypotheses may turn out to be more or less useful in a new situation, and as a result of judgement in the new situation, values may evolve, or new values may emerge that may carry over to new situations in turn.

Values are *(to some extent) shared in society*. Values are not just individual tools that we use to evaluate situations, but they are, at least to some extent, shared in society. They form, as it were, a shared background, based on interpretations from the past, by which we interpret and judge new situations. In this sense, they also have a hermeneutic function. This is not to say that people necessarily agree on all values or how to interpret them in a new situation, but they nevertheless provide a shared starting point for new inquiries. Since people have different (past) experiences, their interpretations of values may also be different, certainly for groups that have different experiences than humans more generally.

### Functions of Values

Now that we have explained our definition of value in more detail, let us focus on the functions they may fulfil as “evaluative devices” in evaluation and inquiry. A first function is that they may help to discover what is (morally) salient in a situation. That is to say, in terms of inquiry, they help to translate an indeterminate situation into a problematic one, and to identify one or more (moral) problems. It should be stressed that this is not just a matter of applying existing values to a new situation but rather involves an interpretation of both the (existing) values and the new situation, in their relation. This interpretation helps to see what is morally salient in a situation but at the same time, it will involve a (re)interpretation of pre-existing values in the new situation.

In this sense, values carrying over from previous situations have the same role as what Dewey calls prizings, which are based on an immediate reaction to a situation. Both provide the raw material for evaluation, but they do not determine our judgement about what ought to be done. This brings us to the second function of values, namely to help normatively evaluate situations. Values do so not by being applied in any straightforward sense to a situation, but rather as input for evaluation, carried over from earlier experiences, and so helping to interpret and normatively judge a situation.

A third function of values is that they may also provide clues or guidance for action. Since we conceive of values as evaluative and not deontic, they are not directly action-guiding. Nevertheless, they may help to suggest certain actions. Or in terms of inquiry, they may help to devise possible courses of actions that transform the indeterminate situation into a determinate one. For example, a value like privacy would suggest a certain way to design COVID-tracing apps, e.g., not based on a central database but only storing information locally and using, for example, Bluetooth to trace with whom infected people have been in contact.

A fourth function of values is that they may help to judge whether an indeterminate situation is indeed transformed into a determinate one, as the end-point of inquiry. Again, it should be stressed that this judgement is not based on pre-given unchangeable values, but that the values themselves may change during inquiry. So the judgement is holistic in the sense that it at the same time judges the situation, and its transformation, as well as the values at stake.

Finally, values may also have a justificatory function. They may help to justify certain choices or courses of action. However, unlike for example in moral realism, they do not do so by being foundational and unchangeable, but rather by helping to embed our judgements in past experiences and by being shared. Values, as it were, form the shared hermeneutic background against which we interpret and judge situations and actions. Values thus fulfil a justificatory role by helping to relate the judgement on the case at hand to prior judgements, and judgements of others, not unlike coherentist accounts in which justification is based on coherence within a larger web of moral beliefs and judgements (cf. Van der Burg 2003).

## The Dynamics of Value Change

Let us now turn to the most important part of our account: how to account for value change? We propose to distinguish three basic dynamics here: value dynamism, value adaptation, and value emergence.

The first dynamics is what we call value dynamism. This is probably the most common situation. These are cases of inquiry (and evaluation) which involve existing values (understood as evaluative devices in the earlier discussed sense) as well as valuings (understood as immediate reactions to a situation). As we have seen, both values and valuings are a starting point for evaluation, but they do not determine the outcome of evaluation. A situation may be valued by an agent in a certain way but upon reflection in inquiry, it may turn out not to be valuable in that way. Similarly, certain values may be used as hypotheses to deal with the situation but they may turn out not to be helpful in the particular situation.

As an example, consider the following simple case. Jane is very hungry and is going to have lunch with her brother Bob. There are, however, only four slices of bread for her and Bob to share. As she is very hungry, her immediate valuing of the situation might be that she wants to eat them all. However, she also brings some generalized values as hypotheses to the situation, for example, the value of “sharing” or “justice.” On the basis of these values, she realizes that she cannot just take all the bread. The tension between the immediate value and the generalized values triggers her to inquire what she should do, and from this inquiry, she concludes that in this specific case, she can take three pieces of bread, as she finds out she is more hungry than Bob, and had she been in Bob’s situation, she would have been satisfied with just one piece of bread. In this example, value dynamism results in a (re)interpretation of the value of justice, or sharing, for the situation at hand.

As the example suggests, this reinterpretation may be due to an individual inquiry, in this case by Jane, but already in this simple example, it would make sense to understand inquiry as a collective process, as it would make sense for Jane to involve Bob in her inquiry rather than to conclude one-sidedly that he can do with one piece of bread. Our interest here is merely in such collective cases of value change.

**Fig. 1:**
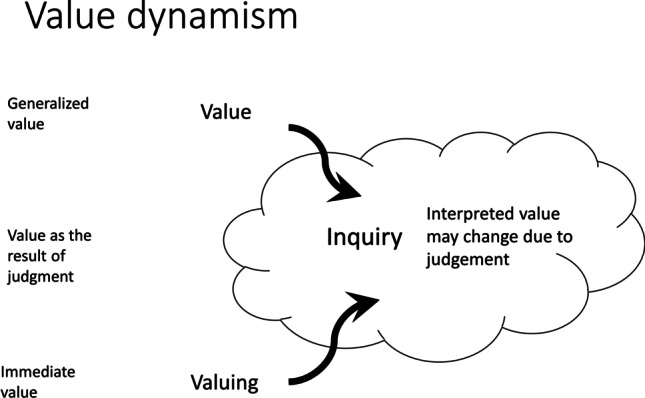
Value dynamism. The terms on the left refer to the corresponding notions of value in Dewey’s writings

As the example illustrates, most of the time, values do not provide a straightforward guide on how to judge situations but instead they need to be (re)interpreted for the specific situation. We call this dynamics *value dynamism*, as it implies a dynamic interpretation of the values in the given situation through inquiry. Figure 1 summarizes this dynamics.

**Fig. 2:**
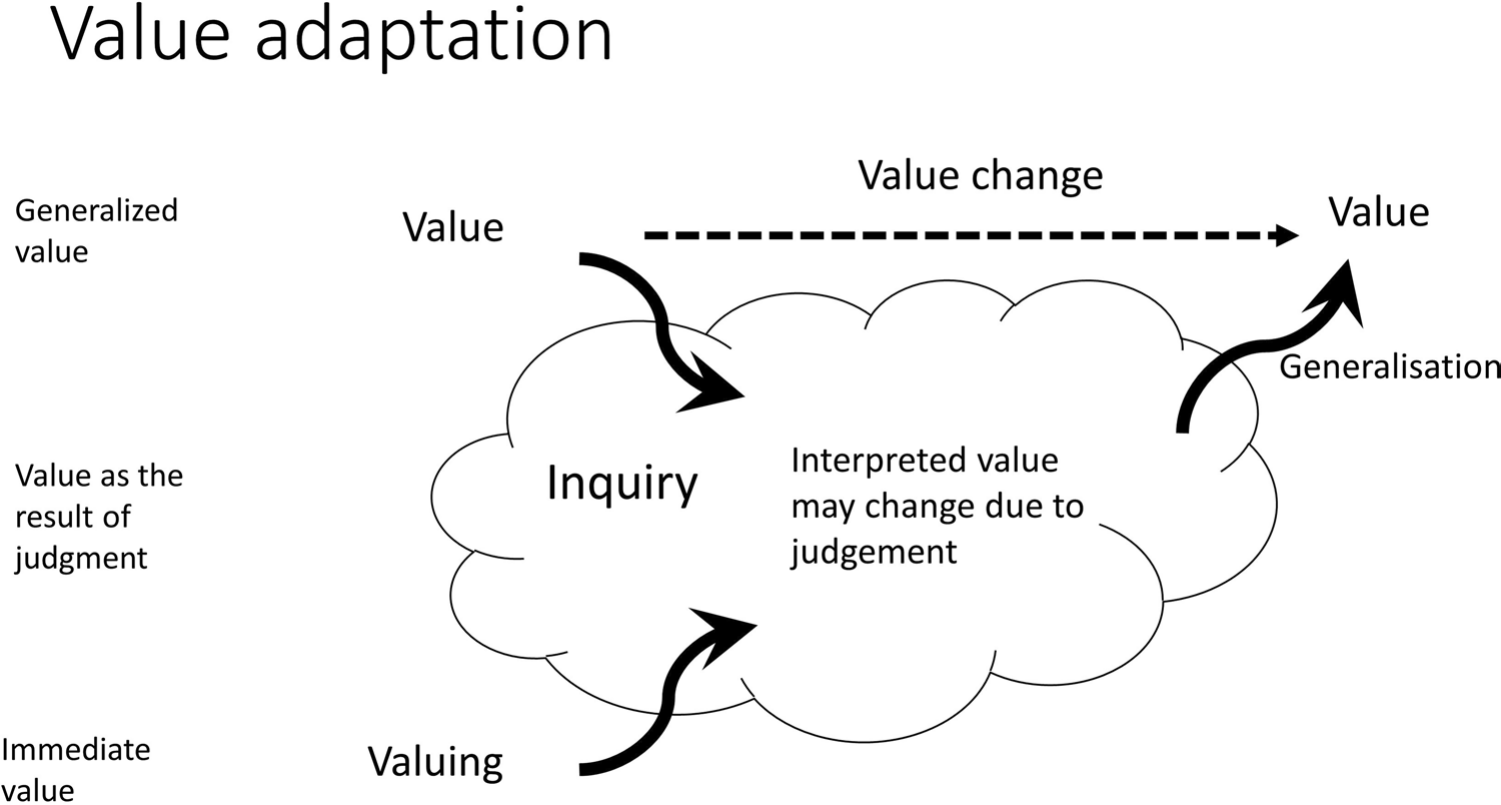
Value adaptation: the reinterpretation of value that is due to value dynamics becomes generalized and results in a change at the generalized level. The terms on the left refer to the corresponding notions of value in Dewey’s writings

The value dynamism in this case resulted in a new interpretation of justice for the case at hand. This reinterpretation may or may not carry over to new situations. If it remains restricted to the case at hand, and henceforth do not imply a change in values understood as “evaluative devices,” i.e., at the level of “generalized value,” we speak of value dynamism. However, if it carries over to new situations, we propose to speak of *value adaptation*. This situation is depicted in Fig. 2, where we witness a more structural change in the value at the general level. We suggest that such changes typically do not happen due to direct changes at the level of generalized values but rather emerge from inquiries where these values were used, and from which a change in the value emerges. Such changes would typically require a larger number of inquiries by different people that show a similar pattern in how a value is (re)interpreted.

As an example, take again the case of Jane. We can imagine that Jane encounters a variety of situations in which she, upon reflection, finds out that in that situation, justice should not be understood as equal distribution, but as a distribution proportional to each other’s needs, not just for bread slices but for the distribution of other goods as well, and others might in their inquiries come to similar conclusions. It is well conceivable that at some point, this leads to a reinterpretation of the value of justice at the more generalized or collective level, so that the generalized value changes from an understanding in terms of equal distribution to an understanding in terms of proportional to needs. We call this dynamics *value adaptation*.

The example might — somewhat simplistically — suggest that value change consists in the replacement of one (interpretation of a) value by another, but values can also come to co-exist at the societal level. For example, rather than “distributive justice as equality” being replaced by “distributive justice according to needs,” the two interpretations, or values, might come to co-exist in society; for example, because some people subscribe to one interpretation and others to the other. However, they may also co-exist as generalized values or cultural resources to which all people have access and which they can use in their local inquiries.

Yet, another case of value change might be that the values themselves, or their interpretations, do not change, but rather the relative importance that people ascribe to them, for example, because they regularly run into indeterminate or problematic situations that require these values. For example, empirical research suggests that in the initial phase of the COVID-19 pandemic, health and safety became relatively more important in society compared to such values as economic welfare, democracy, privacy, and socio-economic equality; later during the pandemic, this (punctuated) value change was somewhat cancelled out (Van de Poel et al., (n.d.)).

**Fig. 3:**
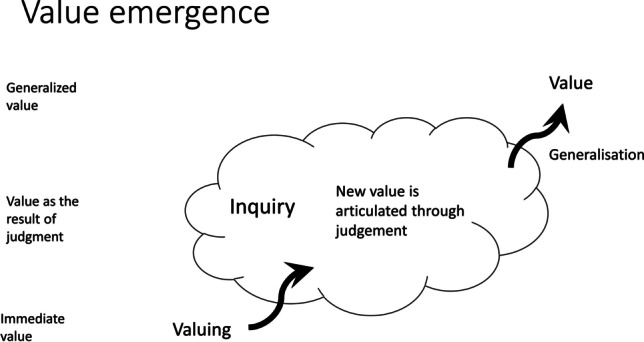
Value emergence: in some cases, existing values may be irrelevant (rather than absent) and the articulation of a new value in judgement may be generalized and so result in a change at the generalized level. The terms on the left refer to the corresponding notions of value in Dewey’s writings

While value change can take many forms, we here want to draw attention to a third theoretical possibility in addition to what we have called value dynamism and value adaptation. We call this possible pattern *value emergence* and it is depicted in Fig. 3. In this case, a value emerges from inquiries about indeterminate situations *without the new value being an adaptation of a pre-existing value*. One might wonder whether, and how, this is possible. One possible objection might be that we always judge new situations in the light of pre-existing values as they form the hermeneutic background against which we interpret and judge new situations. Indeed, above, we said that one of the functions of values is to discover what is morally salient in a situation and to translate an indeterminate situation into a problematic one as the first step in inquiry.

However, we deliberately did not say that in order to recognize an indeterminate situation, we already need values. In line with Dewey’s thinking, we conceive of an indeterminate situation as having a certain urge or unease that does not require pre-existing values to recognize it. Of course, once inquiry starts, pre-existing values may and will be tried to turn the indeterminate situation into a problematic one. However, as hypotheses, pre-existing values may not be successful in the sense that they are not helpful in eventually transforming the indeterminate situation into a determinate one. So while in a sense, there are always pre-existing values, they may turn out to be irrelevant or unproductive for the indeterminate situation at hand. While such situations may be rare, they are certainly not inconceivable.

Think of the hypothetical case of a society in which justice is not yet a value. And think of the kind of indeterminate situation that Jane found herself in in the first example. Hungry people may be inclined to keep all food for themselves if there is not yet a generalized value like “sharing” or “justice.” If this happens time and again, it may lead to other people becoming dissatisfied or even angry. Not only may disadvantaged people get angry, people may alternately be in a more advantaged or disadvantaged situation with respect to food and find out that “sharing” might be a better solution to such indeterminate situations than everyone just keeping their own food. This solution might first be practiced locally and occasionally, but if it offers a better way to deal with the indeterminate situation, it might spread and get generalized and lead to the emergence of the new value of “sharing” or justice.

This process of value emergence is schematically shown in Fig. 3, which suggests that values in such cases emerge in two distinct steps. The first one is that in inquiry, we reflect on our valuings (or prizings), which provide a direct or immediate reaction to the situation at hand, and in doing so, we develop a judgement about what is desirable in the situation at hand. In the example, different valuings or prizings may be going on. First, there is the valuing of the hungry person who has the food and may be inclined to eat it all. Then there is the (immediate) valuing of the other party, who might get angry that the first person keeps all the food for herself. These conflicting valuings give raise to an inquiry, and it is in this inquiry that a new value may arise, like the value of sharing or justice. This inquiry thus results in a new value that did not yet exist before; at this stage, the new value, however, is not yet generalized, that is to say, it does not yet carry over from past experiences nor is it shared. It may perhaps be better called at this stage a proto-value, in the sense that it is a value-in-the-making.

The second step is the generalization of this proto-value into an abstract value that can also be used in other situations disconnected from the situation in which it arose. It may be assumed that this generalization step will only occur if we are confronted not just once but more often with situations that require a certain proto-value for their resolution, and if others run into similar situations in which a similar proto-value is articulated. In fact, in our hypothetical example, it seems quite likely that a society in which justice is not yet a value would regularly run into distributive problems that cause anger or dissatisfaction, and that the value of distributive justice may emerge at some point to better deal with such situations.

## Technology and Value Change: Three examples

In the previous section, we have proposed a conceptual framework for understanding value change based on pragmatism. In this section, we will show how we can make this framework operational for cases of technology-induced value change. We will discuss three cases, which roughly correspond to the three dynamics of value change distinguished in the previous section. We do not claim that these three cases exactly fit the three distinguished dynamics (i.e., value dynamism, value adaptation, and value emergence) but rather that these three dynamics, and more generally, our conceptual framework is helpful for understanding these three cases. For each, we will present a pictorial representation that has the same elements as Figs. 1, 2, and 3 above.

It should be noted that while value change may occur due to individual inquiries, we here focus on collective inquiries that were triggered by new technological developments, as we believe this might help us to better understand how technology may induce value change not just at an individual but also at a collective level, which is usually seen as the thing to be understood in the literature on technology and moral change (e.g., Swierstra, 2013). In the next section, we will use these cases to reflect more systematically on how and why technology may induce value change.

### Google Glass

The introduction of Google Glass in 2013 prompted discussions regarding the value of privacy. The mixed reality goggles equipped with camera and connected to cloud services promised to overlay physical reality with a virtual one, letting the user simultaneously be active in both worlds. Google paid a lot of attention to the privacy of Glass’s users, suggesting that “Our vision behind Glass is to put you back in control of your technology,” including the user’s data (Wayback Machine, 2015). Privacy as Google understood it here is a value that carries over from previous situations as hypothesis.

However, the people around Glass wearers could not relate to the privacy-as-control-of-information conception because it did not fit the new practices that the device enabled. Instead, they referred to Glass as “the end of privacy as we know it” (Kudina & Bas, 2018, 130), but this did not mean that privacy had become irrelevant, but that it needed to be redefined in reaction to the new type of indeterminate situations created by Google Glass.

**Fig. 4:**
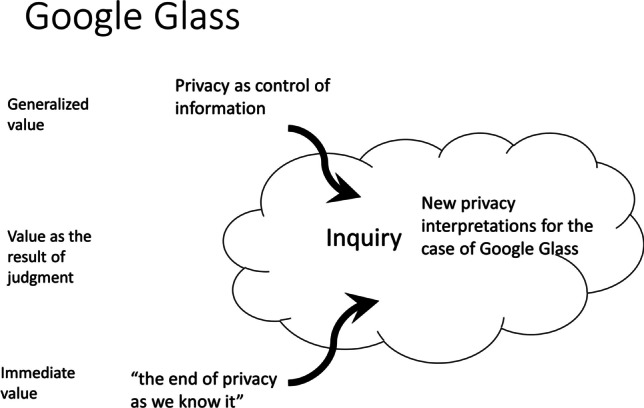
Value change dynamics in the Google Glass case

In absence of visual signals that could identify whether the Glass user was recording a video or taking a photo, people became suspicious of Glass wearers in public spaces, on dates, in gym locker rooms, cinemas, and on other occasions. In response, people devised multiple new understandings of privacy that helped them deal with the lack of control over their information in the age of Glass. Such new privacy conceptions addressed the social and relational dimensions of privacy regarding identity building, civil inattention, sharing one’s experiences online, blurring the line between remembering and forgetting, etc. (Kudina & Bas, 2018; Kudina & Verbeek, 2019). In these processes of inquiry, privacy appeared as a multi-layered, porous, and dynamic value (Fig. 4 ).

**Fig. 5:**
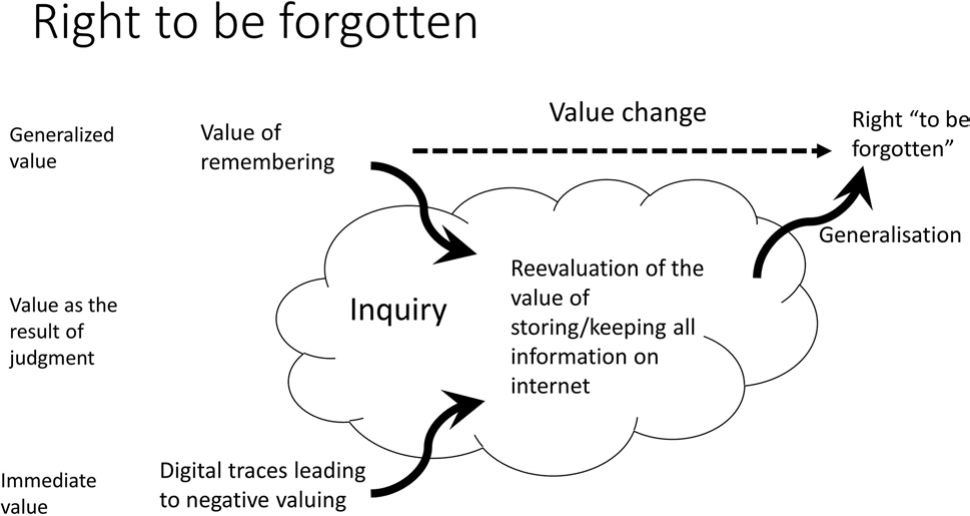
Value change dynamics in the “right to be forgotten” case

**Fig. 6:**
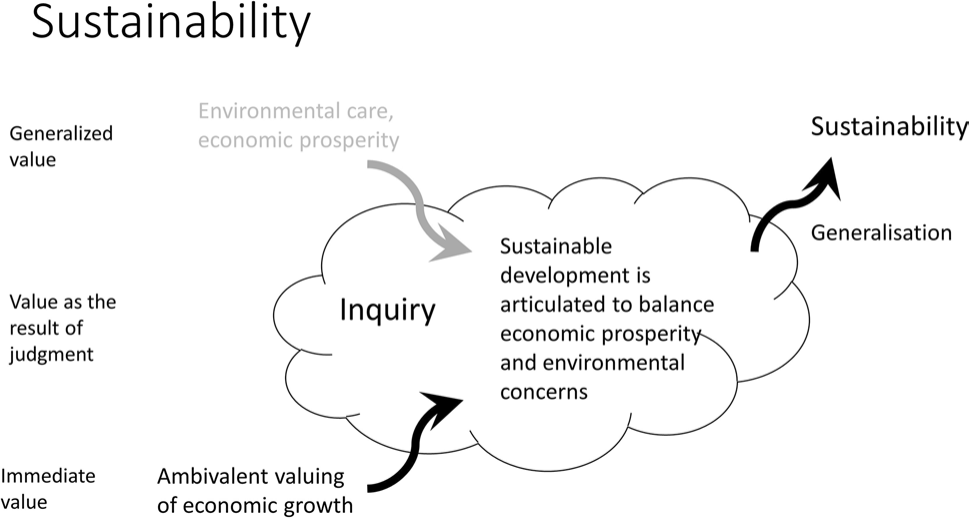
Value change dynamics in the sustainability case

In this example, we mainly witness what we have called value dynamism. When confronted with the inability of the privacy-as-control-of-information conception to address the newly challenging practices that Glass enabled, and on basis of immediate, pre-reflective valuations (i.e., “The end of privacy as we know it”), people started to inquire how to understand the value of privacy when faced with the device. As a result of this exercise, several new privacy interpretations emerged as more fitting guides for action in the world of which Glass is a part. Thus, privacy still continued to play a guiding role in dealing with this technology, albeit in a reconceptualized manner and better fitted to address the situations at hand. In as far as these new understandings of privacy remained local and specific for Google Glass, the observed dynamics may be described as value dynamism; in as far as it has spilled over to the more general debate about and understanding of privacy, for example in relation to digital technologies, we may speak of value adaptation.

### The Right to Be Forgotten

Pervasive Internet connectivity and digital technologies have brought about many opportunities, inconceivable in the past: to connect over distance, to access any information anytime from anywhere, and to access education and medicine regardless of geography and financial means, etc. Digital technologies and the Internet were premised on the value to remember everything, forever. At the same time, Internet-based communication implicitly and explicitly wove the canvas of our social and private lives, fostering an expectation of constant availability and accessibility of information. Internet became an everlasting digital repository of memories and digital footprints, where nothing is forgotten and any digital trace from any point of one’s past can have lasting repercussions in the present. Over time then the use of Internet led to new indeterminate or problematic situations, for instance, being fired from a teacher’s position because of an old Facebook photo with alcohol bottles or not being able to find a job because the first Google search line of you mentions your long-served felony (Mayer-Schönberger, 2009). The existence of irremovable digital traces has led to new negative and immediate valuings by people and a feeling that something was wrong with current practices. This led to inquiries at an individual and collective level and people asked for their information being removed from servers.

The year of 2014 marked an important legal and moral change in the European context, witnessing an introduction of “A right to be forgotten” that manifested people’s value to not be defined by their digital representation and the right to demand removal of the digital material that misrepresents them. We classify this example as value adaptation because it depicts how multiple localized instances of value dynamism over time result in a larger structural change and reaffirm a previously underexposed value, while reinterpreting its meaning to address the new technological challenges. Several societally prominent (e.g., Google Spain v. AEPD, Costeja González [2014]) and individual moral disagreements and inquiries had to take place before a larger change in value emerged.

Thus, in about 20 years of the active use of digital technologies and the Internet, we see first the articulation and then the institutionalization of a value that seems diametrically opposed to the initial value of wishing to remember all the information online (Fig. 5). Of course, the new value and the old value may to some extent exist next to each other, as they may apply to different pieces of information for different people.

So one may debate whether this is really a case of value adaptation, in which an existing value is adjusted, or a case of value emergence, in which a new vale emerges. One might even argue that it is a case of value dynamism.^4^ That is to say, one might argue that the value of forgetting and “the right to be forgotten” is not specific for digital technologies and had been established before. For example, the UK Rehabilitation of Offenders Act 1974 enables some criminal convictions to be ignored after a rehabilitation period.^5^ Similarly, case law concerning celebrities suggests that the need to balance privacy and the right of the public to be informed was not an entirely new challenge. Nevertheless, the digital age seems to require finding a new balance (Yanisky-Ravid & Zion Lahav, 2017), and therefore, we witness at least a form of value dynamism, as existing balances of values had to be reinterpreted for the internet and digital age.

What is important in understanding the dynamics of this value change is that the initial value of perpetual remembering could not cope with the unforeseen challenges and expectations of the digital age, which triggered new inquiries and subsequently value changes. We return to this point in the next section.

### Sustainability

In the case of sustainability as a value emergence, we witness how initially localized indeterminate situations acquire global scale and become recognized problematic situations that merit both local and global solutions. Since the appearance in 1987 of the Brundlandt report on sustainable development (World Commission on the Environment and Development 1987), sustainability has emerged as a global concern and gained a prominent place in the moral vocabulary. Nevertheless, concerns regarding what we now call “sustainability” have consistently appeared throughout human history. In ancient times, Plato and Columella worried about the dangers of farming, mining, and “maintain[ing] the ‘everlasting youth’ of the earth” (Du Pisani, 2006, 85). The term itself was coined in 1713 in the context of replenishing forest resources in Germany (Van Zon, 2002). But it was from the twentieth century onward that the challenges of global industrialization and the rapid technological innovation manifested most prominently. The value of sustainability was thus long in the making, initially as an ad hoc solution and appeal in the local indeterminate situations and gradually, becoming a generalized collective inquiry recognizing that something is collectively morally at stake. Here, we can speak of sustainability as an emerging proto-value, becoming recognized but still rather abstract.

Until the 1960s, economic and technological development had been considered largely in terms of linear progress, development, efficiency, and prosperity. However, as Du Pisani contends, “After two global wars it was evident that, particularly in the moral domain, there was also a downside to scientific and technological advances […]. By the 1970s the idea of continuous progress was losing much of the fascination” (2006, 89). The Hiroshima and Nagasaki atomic bombings, the destruction of wildlife by the indiscriminate use of pesticides (Carson, 1962), Chernobyl nuclear power plant explosion, and many other technologically-prompted disasters helped to change the technological narrative to include considerations over the risks and harms they might induce. As the knowledge about ecological degradation consolidated globally, the spread of television helped to popularize it, supported by vivid images. Within these challenges, one could also trace an emergence of a new type of concern and orientation, imbedded in a new value of sustainability. This is where the generalization step occurred, bringing the proto-value of sustainability under collective scrutiny. The morally problematic situations that call on sustainability kept appearing not just once and locally but became reoccurring at a global scale, until sustainability emerged as a generalized orienteer called on to address such determinate problematic situations (Fig. 6).

Following Mitcham (1995), the new value of sustainability aimed to address the conflict between a possibility of development, while acknowledging the limits to growth: there can be no development without conservation. A new aspect of the previously existing notion of sustainability also appears in this time. Sustainability not only exceeds the immediate temporal considerations and individual instances but also has a global scope in fostering a continuous harmonious coexistence of people with nature, while accounting for the novel socioeconomic developments (World Commission on the Environment and Development 1987).

The value of sustainability is an example of value emergence because even though one may trace its origins throughout human history, it was not until the twentieth century that it emerged as a consolidated shared concern and guidance addressing the rapidly changing and developing world. Its emergence did not occur in a moral vacuum and was not unproblematic. The value of sustainability crystalized against the morally salient background of the technological catastrophes, the growing divide between developed and developing nations, and the clashes of efficiency versus safety, perpetual development versus preservation. Neither of the existing values could singlehandedly address the increasingly global scope of sustainability, although they all served as first steps in the critical inquiry that underwent its development and turned a morally indeterminate situation into a determined, problematic. Similarly, the step towards generalizability of the value of sustainability did not happen overnight and is still not finished, albeit increasingly recognized globally. Initially recognized and promoted in the areas that suffered the immediate aftermath of the technologically environmental catastrophes, at present, few countries remain unaffected by the phenomena of global warming and climate change. Thus, at the beginning of the twenty-first century, the value of sustainability became an almost globally embraced moral guideline and evaluative device in crafting a harmonious coexistence of people with their — increasingly technological — environment.

## How Technology May Trigger Value Change

Our framework is useful for specifying and understanding cases of technology-induced value change, as we saw in the previous section. We now want to reflect on what the driving force is behind such a technology-induced value change. We submit that based on the pragmatist framework, a main driving force behind value change is the creation of new indeterminate situations. As we have seen, for Dewey, such situations are characterized by a certain feeling of unease, discomfort, or the impression that something is wrong. It is these feelings that trigger inquiries that can lead to value change.

More specifically, we claim that one of the main ways in which new technology can induce value change is by creating new indeterminate situations. These new indeterminate situations, in turn, may lead to new valuings (i.e., new immediate value) and it is the tension between these valuings and generalized values that triggers the process of inquiry, and this — in turn — may lead to new moral judgements that can establish new or adapted values.^6^

We see this dynamics in all three cases discussed in the previous section. In the case of Google Glass, the new device created new indeterminate situations, which triggered a valuing like “the end of privacy” but also feelings of discomfort when Glass was used, for example, in a restaurant during a dinner. These valuings were at tension with the generalized value of privacy-as-control-of-information. Similarly, in the other two cases, technological developments created new indeterminate situations, which led to valuings like discomfort with the storage of information on internet or worries about environmental effects of technology and economic development, which were at tension with current generalized values.

It may be hypothesized that such processes of technology-induced value change will often have an iterative or recursive character. That is to say: in response to an indeterminate situation, new values may be articulated and new technologies may be (experimentally) tried out in an attempt to solve the indeterminate situation, but the solution might not or not fully work in practice, so new indeterminate situations might arise that require a response again, and so on.^7^

To understand this dynamics, it is important to see that it has both a more “objective” component and a more “subjective” one. The objective component is that technologies may create new indeterminate situations, independent from people’s desires, beliefs, and wishes.^8^ Also, whether, and to what degree, new values and technologies solve indeterminate situation is not primarily dependent on human values and beliefs but depends to a large extent on the external world. Dewey (1925) expresses this dependence on the external world by saying that values have an objective referent.

But in addition to this more “objective” component, there is also a subjective one in the sense that only when, and in as far as, indeterminate situations trigger a feeling of unease and new immediate values through valuing, they can lead to inquiries and result in value change. Here subjective, psychological factors clearly play a major role, although it should be stressed that valuing, and new immediate values, is mainly a trigger for inquiry, which is a reflective, interpretative, and hermeneutic process. This means that on this understanding technology does not *directly cause* value change; rather, it creates the conditions (i.e., new indeterminate situations) that can trigger new inquiries, and it is these interpretative inquiries that can lead to value change.

We now want to compare this account of technology-induced value change with three other existing accounts in the literature, namely Swierstra’s work on technomoral change (Swierstra, 2013; Swierstra et al., 2009), Nickel’s discussion of the role of moral uncertainty (Nickel, 2020), and Baker’s work on moral revolutions (Baker, 2019).

Like our account, Swierstra’s account of technomoral change draws on ideas from philosophical pragmatism. He particularly conceives of technology as a potential disruptor of existing moral routines. In doing so, he distinguishes between “cold” morality, as the routines we normally take for granted, and “hot” ethics, as the debate that may be triggered when existing moral routines are disrupted, for example, by technology. Swierstra’s moral routines seem somewhat similar to what we have called generalized values. Our analysis then is in line with the general point of Swierstra that technology may disrupt or put in question what he calls moral routines and what we have called generalized values. However, we believe that our analysis is somewhat more precise and detailed. By analyzing value at three levels, we are better able to understand the disruption and its effect, namely as giving raise to *new indeterminate situations,* which trigger *new immediate values* that are at tension with existing general values. This tension triggers *new inquiries* and depending on how such inquiries evolve, they may result in either value dynamism, value adaptation, or value emergence.

Nickel (2020) draws attention to the role of moral uncertainty in processes of moral disruption. He posits that moral disruption (through technology) typically comes with moral uncertainty. This seems to be consonant with our account in the following sense: on our account, moral uncertainty may be understood as a response to a new indeterminate situation in which existing generalized values no longer provide a proper response. However, our account suggests that value change may not require moral uncertainty. It may be enough that there is a tension between immediate value and generalized value, that tension may lead to moral uncertainty but it may also manifest itself as controversy or an anomaly.^9^

This brings us to Baker’s account of moral revolutions. Baker proposes to understand moral revolutions in analogy with Kuhn’s account of scientific revolutions. One of his suggestions is that moral revolutions may at some point occur due to the accumulation of moral anomalies. Baker understands an anomaly as “something that does not fit an established paradigm” (Baker, 2019: 42). Our account suggests a somewhat more precise understanding of an anomaly, namely as a persisting or unresolved tension between immediate values and generalized values. In other words, a moral anomaly arises if a tension between immediate values and generalized value cannot be solved, at least not for the time being, through a (local) inquiry. Our account not only helps to understand how moral anomalies may arise, but it — additionally — can explain how technology can play a role in it, i.e., by creating new indeterminate situations that lead to new valuings at tension with existing values, which if unresolved may become moral anomalies.

## Conclusions

We have offered a pragmatist account of value change. On this account, values are primarily understood as evaluative devices that carry over from earlier experiences and are (to some extent) shared in society. We have further argued that the dynamics of value change may be understood by a conceptual framework that is inspired by Dewey’s distinction between “immediate value,” “values as the result of inquiry,” and “generalized values.” This framework may help to understand individual value inquiries, as well as collective value inquiries resulting in value change. We have argued that within this framework, we might distinguish between three ideal–typical processes of value change, which we have called value dynamism, value adaptation, and value emergence.

We have further shown that this framework may not only help to understand individual cases of value change triggered by technological developments but also goes some way in explaining how technology may induce value change, i.e., by creating new indeterminate situations, and we have shown that our analysis is in line with some of the insights offered by authors such as Swierstra, Nickel, and Baker, while offering a more comprehensive conceptual framework. Our account also suggests that value change may sometimes be needed to adequately deal with new indeterminate or problematic situations.

On our account, value change is not primarily driven by changes in moral beliefs or human desires, although these also play a role, but rather by the emergence of new types of indeterminate situations. These changes are in a sense “objective” and “external” to us, although they are of course in many cases the result of human activities and choices. Typically, the new indeterminate situations are the unintended, and often unforeseen by-product of human activities, and values may need to change, or new values may need to emerge, to deal with these new indeterminate situations. As Dewey already foresaw, this process is probably endless, as each transformation of an indeterminate situation into a determinate one, even if temporarily successful, may also, unintentionally, produce new indeterminate situations, and hence the need for new inquiries and values. It is through such continuous inquiry that we can gradually improve the human condition.

## Data Availability

Not applicable.

## Footnotes

1 Our reading of Dewey’s value theory below is largely based on Dewey (1915; 1918; 1922a; 1922b; 1925; 1929b and 1939). We also relied on Anderson (2019).

2 Dewey (1922b, 329) says that there are six significations of value, which consist of the three distinguished here and for each an immediate (or isolated) version, and a contributory version, which appears to map on the final/instrumental value distinction. We leave out the second distinguishing dimension here and focus on the first dimension.

3 Our description of Dewey’s notion of inquiry is largely based on Chapter 7 “The Pattern of Inquiry” in Dewey (1938).

4 We thank an anonymous reviewer for this suggestion and pointing us to case law concerning celebrities and the Rehabilitation of Offenders Act 1974.

5 https://www.legislation.gov.uk/ukpga/1974/53 Accessed 17 January 2022.

6 Remember that, for Dewey, valuing is immediate and unreflective. It is the first step in valuation (or inquiry), which has also a reflective stage. One might hypothesize that if valuing, and the immediate value following from it, is not at tension with generalized values, no inquiry is needed; so inquiry seems particularly triggered if there is a tension between immediate values and generalized values.

7 We do not claim that this pattern is unique to technology-induced value change. Indeed, we also expect the cycle problem — potential solution — new problem to occur for other types of value change, but we restrict our discussion here to technology-induced value change.

8 Obviously, technologies have no free will. So the creation of new indeterminate situations is due to (human, epistemic) uncertainty and ignorance and the indeterminate character of technology development (Van de Poel 2017).

9 Nickel seems to recognize this; on his account, people may deal differently with the moral uncertainty that comes with moral disruption, some may simply deny it or try to suppress it “so that the public face of moral uncertainty will often be that of disagreement and controversy, rather than perplexity” (Nickel 2020: 267).
